# Vanishing Lung Syndrome: Compound Effect of Tobacco and Marijuana Use on the Development of Bullous Lung Disease – A Joint Effort

**DOI:** 10.7759/cureus.1530

**Published:** 2017-07-31

**Authors:** Shimshon Wiesel, Faraz Siddiqui, Tahir Khan, Sami Hossri, Dany El-Sayegh

**Affiliations:** 1 Internal Medicine, Staten Island University Hospital, Northwell Health; 2 Pulmonary and Critical Care Medicine, Staten Island University Hospital, Northwell Health

**Keywords:** marijuana, pulmonology, bullous lung disease, vanishing lung syndrome, critical care, ards, smoking, tobacco

## Abstract

Marijuana use has been increasing across the United States due to its legalization as both a medicinal and recreational product. A small number of case reports have described a pathological entity called vanishing lung syndrome (VLS), which is a rare bullous lung disease usually caused by tobacco smoking. Recent case reports have implicated marijuana in the development of VLS.

We present a case of a 47-year-old man, who presented to our hospital with shortness of breath, fevers and a productive cough. On physical exam, he was tachypneic with audible stridor and absent right sided breath sounds. Laryngoscopy showed a retropharyngeal abscess, and chest radiography showed a possible right pneumothorax. Chest computed tomography (CT) showed bilateral bullous emphysematous lung disease with a giant bulla occupying most of his right lung field. He was placed on mechanical ventilation and treated with broad spectrum antibiotics in the intensive care unit, where he developed acute respiratory distress syndrome (ARDS). He continued to decline, and developed disseminated intravascular coagulation, after which he succumbed to his disease.

## Introduction

Vanishing lung syndrome (VLS) is a radiographic entity first described in 1987 as giant bullae displacing normal lung tissue and occupying at least one-third of the hemithorax. VLS is usually associated with smoking tobacco, but marijuana has also been implicated in recent literature. Legalization of recreational use of marijuana in certain states, and growing marijuana use in the adult population may lead to an increase in the incidence of VLS. Misconception about the harmlessness of marijuana should be a public health awareness issue. We highlight this point by presenting a rare case of VLS in a marijuana user, complicated by acute respiratory distress syndrome (ARDS), with a brief literature review on the contribution of marijuana to the pathogenesis of VLS.

## Case presentation

**Figure 1:**
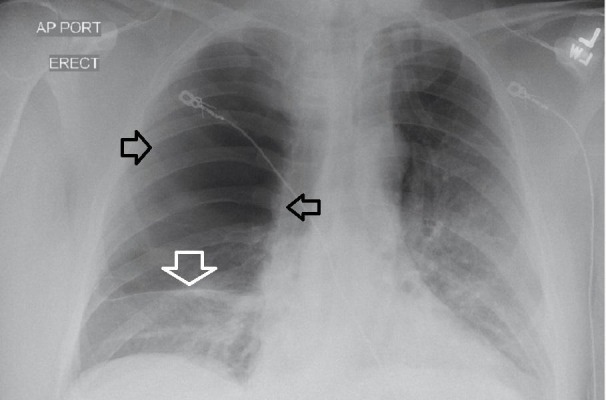
Chest radiograph on admission. Lung parenchyma is not apparent in the right hemithorax consistent with pneumothorax (black arrows). However, this is bullous disease occupying most of the right hemithorax. The remaining right lung tissue at the bottom of the lung field has an opacity (white arrow).

**Figure 2:**
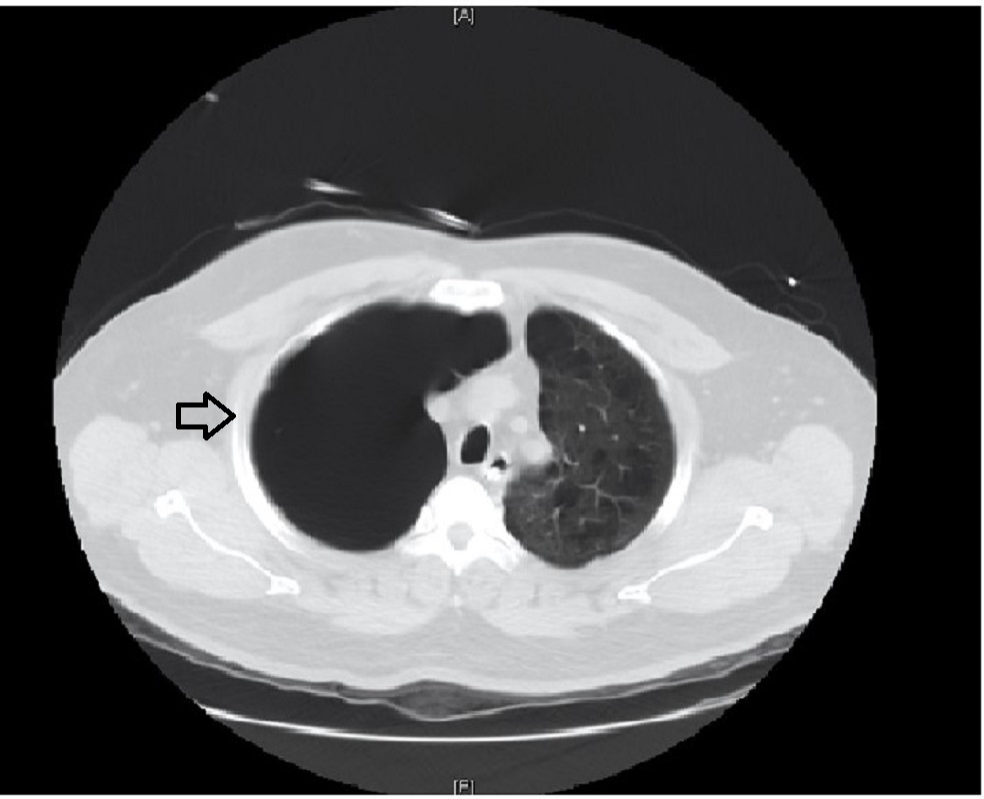
Axial computed tomography (CT) of the chest on admission. A large bulla (black arrow) occupying the right hemithorax.

**Figure 3:**
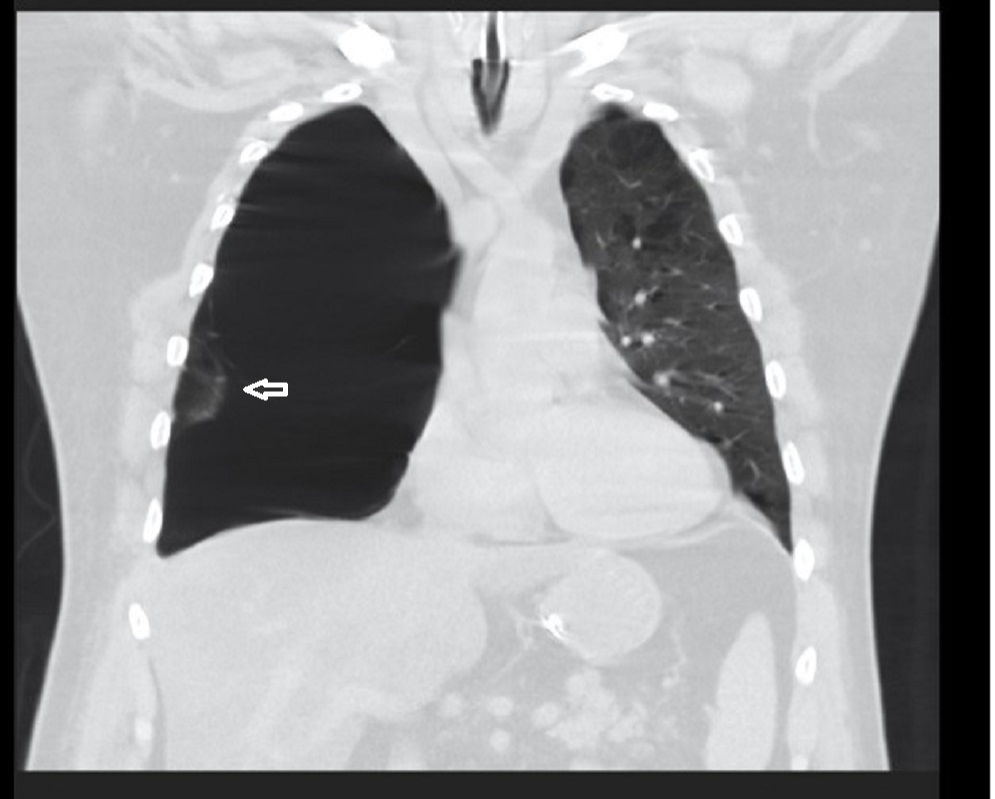
Coronal computed tomography (CT) chest on admission. Reconstituted coronal view of CT chest illustrating bullous disease in the right hemithorax. There is minimal lung parenchyma (white arrow) present, but with bullous changes.

**Figure 4:**
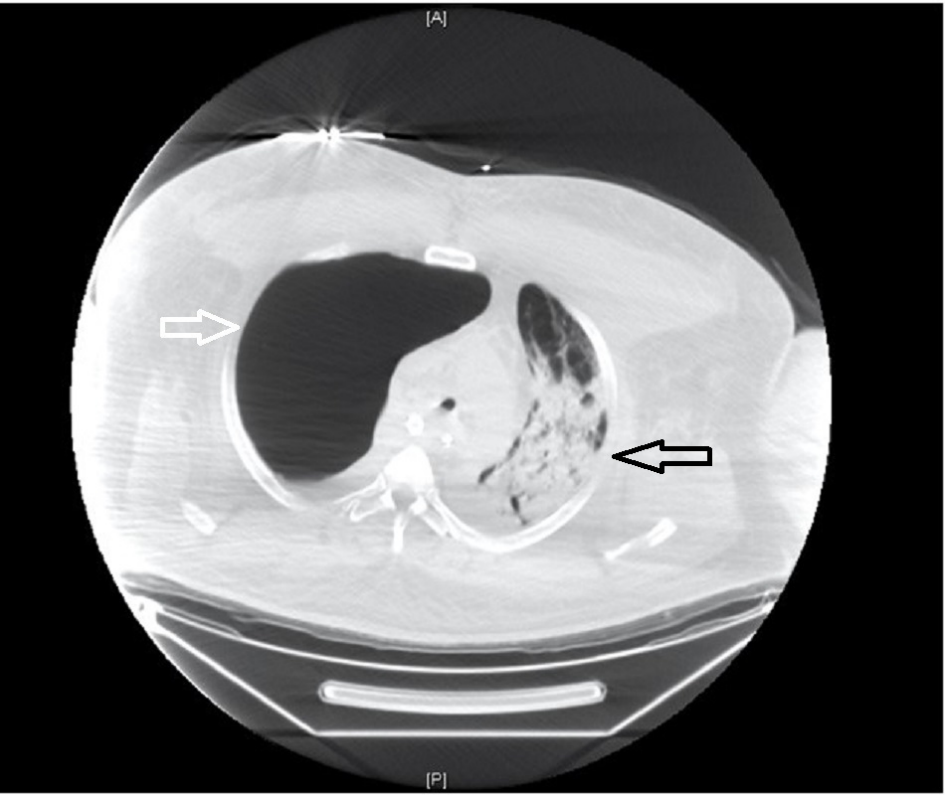
Axial computed tomography (CT) chest showing acute respiratory distress syndrome (ARDS). Axial CT chest again, illustrating right-sided bullous disease (white arrow). There is also interval development of left-sided consolidation consistent with ARDS (black arrow).

**Figure 5:**
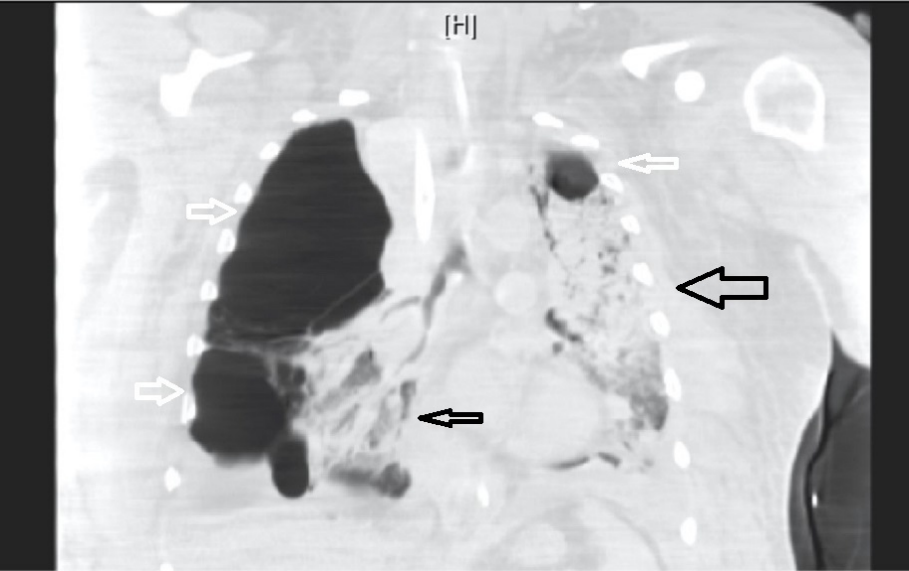
Coronal computed tomography (CT) chest showing acute respiratory distress syndrome (ARDS). Reconstituted coronal CT chest illustrating right-sided bullous disease and left-sided apical bullous disease (white arrows). Also shown is left-sided consolidation with minimal right-sided consolidation consistent with ARDS (black arrows).

A previously healthy 47-year-old man presented to the emergency department with a three-day history of shortness of breath, productive cough with yellow sputum, and fevers. He had a 20 pack-year and 25 joint-year history of smoking cigarettes and marijuana, respectively. He denied alcohol use. He had no significant occupational or animal exposures. On examination, vital signs were remarkable for a heart rate of 138 beats per minute, respiratory rate of 23 breaths per minute, and a temperature of 101.4 degrees Fahrenheit. Oropharyngeal exam was remarkable for marked pharyngeal edema and erythema. Chest examination revealed audible stridor, decreased breath sounds over the right hemithorax as well as in the upper half of the left hemithorax and left sided rales. The rest of the examination was unremarkable. Laboratory studies showed leukocytosis of 18,800 WBC/mm^3^, hemoglobin of 17 g/dl and a platelet count of 262,000 platelets/mm^3^. Comprehensive metabolic panel, cardiac enzymes, and electrocardiogram were within normal limits. Chest radiography showed a possible right pneumothorax, left sided apical bullae, and bibasilar opacities (Figure 1). Computed tomography (CT) of the neck and chest were done and showed retropharyngeal abscess and bilateral bullous emphysematous lung disease with a giant bullous occupying nearly the entire right lung field (Figures 2-3). Echocardiogram showed normal findings. A diagnosis of giant bullous lung disease with emphysema complicated by retropharyngeal abscess was made based on radiographic findings. Broad-spectrum antibiotics were initiated. Blood and sputum cultures grew no organisms. His alpha-1 antitrypsin levels were normal. He developed septic shock and worsening hypoxemia in the following 48 hours and required mechanical ventilation and vasopressors. Antibiotics were escalated to cover *Pseudomonas aeruginosa* and methicillin-resistant *Staphylococcus aureus*. Repeat CT chest showed worsening left lung consolidation consistent with ARDS (Figures 4-5). He showed no signs of improvement on mechanical ventilation requiring 100% FiO_2_. ARDS protocol mechanical ventilation was started and an arterial blood gas (ABG) showed: pH 7.27, paCO_2_ 94, and paO_2_ 55. Echocardiogram with bubble study was done to rule out intracardiac shunt as a concomitant cause of hypoxemia and did not show bubbles in the left ventricle. Veno-arterial extracorporeal membrane oxygenation (ECMO) was initiated due to severe refractory hypoxemia, septic cardiomyopathy, and severe metabolic acidosis. Unfortunately, he developed disseminated intravascular coagulation (DIC), went into cardiac arrest and could not be revived.

## Discussion

VLS is a rare condition, defined as large bullae occupying at least one-third of the hemithorax. VLS mimics tension pneumothorax radiographically, as seen in our patient, and CT of the chest is the imaging modality of choice to differentiate between the two conditions. Bullectomy is the mainstay of treatment for symptomatic improvement, but does not restore the collapsed lung in most cases. Hence, it is important to know the various risk factors and pathogenesis of VLS in order to prevent its occurrence. Until recently, tobacco smoking was the most common risk factor, along with alpha-1 antitryptin deficiency. Marijuana smoking is emerging as a contributing risk factor for VLS, especially in concomitant tobacco smokers. The National Institute of Health reports that 9.5% of Americans use marijuana, and 30% of marijuana users meet criteria for a disorder. Marijuana is the second most widely smoked substance in the USA after tobacco. According to the 2012 national survey on drug use and health from the Substance Abuse and Mental Health Service Administration, up to 18.9 million people in the USA acknowledged use of marijuana, 7.2% of which were children ages 12 to 17 years of age, and 18.7% of them being young adults, 18 to 25 years of age. As per the 1970 Controlled Substances Act (CSA), possession or use of any quantity of marijuana was an illegal and punishable offense. However, recently in the USA, the medicinal use of marijuana has been legalized in 26 states, and the recreational use is permitted in four states. Changing policies on marijuana use in the USA are expected to increase its usage, and could potentially create an enormous burden on health care costs, especially due to its role in developing respiratory diseases [1]. In our patient, the development of extensive bullous lung disease at a relatively young age, with normal alpha-1 antitrypsin levels, questions the sole role of tobacco smoking as a contributing factor. His concomitant use of marijuana may have acted as a potential catalyst for the premature development of giant bullous lung disease [2].

Marijuana has been proven to have a deleterious impact on lung function, independent of tobacco smoking. Tashkin, et al. demonstrated a significant linear correlation between decreased maximal mid-expiratory flow rate and specific airway conductance with the quantity of marijuana smoked over time [3]. In their study, the prevalence of chronic cough, sputum production, wheezing, and more than one episode of prolonged acute bronchitis during a three-year period was significantly higher in users of marijuana, tobacco, or the combination of both substances. In comparison to tobacco smoking, marijuana has greater adverse effects on large airway function by increasing airway resistance, whereas tobacco smoking damages small airway and alveoli as shown by a decrease in carbon monoxide diffusing capacity (DLCO) with the use of the latter [3]. Hyperinflation of the lungs caused by the damaging effect of marijuana on the large airways causes an increased forced vital capacity (FVC), with limited effect on DLCO [4]. Damage to the large airways may be attributed to the dynamics of marijuana inhalation, with two-thirds larger puff volume, a one-third greater depth of inhalation, and a four-time longer breath-holding time than tobacco inhalation, thereby creating high inspiratory pressures. Additionally, prolonged breath holding causing valsalva pressure contributes to barotrauma leading to bullous disease [3,5]. Marijuana inhalation, regardless of its tetrahydrocannabinol content, bears a greater burden of carbon monoxide (CO) content in the blood and a greater degree of tar deposition in the respiratory tract than an equal quantity of tobacco smoking [6].

The individual use of marijuana and tobacco inhalation causes significant bronchial mucosal histopathologic changes, and their concomitant use appears to have an additive effect [7]. Roth, et al. observed, via video bronchoscopy, a significantly higher number of visible changes of bronchitis such as erythema, edema and increased secretions in populations that are marijuana and tobacco smokers. Their visible bronchial mucosal changes correlated to histopathological changes of hyperplasia of goblet cells, inflammatory cell infiltrates, and submucosal edema [7-8]. Longitudinal observational studies showed a marginally significant dose dependent relationship between cumulative marijuana smoking, and a decline in forced expiratory volume during the first second (FEV1) [9]. A recent epidemiologic study also suggests that a 50 joint-year history of marijuana smoking is associated with a decline in FEV1 [10].

Our patient developed severe bullous lung disease after a relatively short period of time, which can reasonably be attributed to his concomitant use of tobacco and marijuana. The long-term effects of marijuana on respiratory health remain to be determined. Furthermore, the legalization of marijuana for both medicinal and recreational purposes strengthens the need for more research as well as public health awareness initiatives to explore the effects of marijuana smoking. Meanwhile, physicians should have a low threshold to screen patients using marijuana for the development of bullous disease. Once diagnosed, the patient should be promptly referred to a pulmonologist and a cardiothoracic surgeon to be evaluated for surgical intervention at an appropriate time.

## Conclusions

Our case underscores the harmful synergistic effects of marijuana and tobacco use on the development of bullous lung disease. Our patient had advanced bullous lung disease at the time of his diagnosis, which precluded our ability to offer meaningful medical or surgical intervention. The shifting political climate and the legalization of medicinal and recreational marijuana behoove more research and resources to be allocated to understanding its potential health hazards. Often misconceived as a “harmless” substance, marijuana can cause catastrophic damage to the lung and can have grave repercussions on public health with its ongoing legalization.
